# Evolution of tick vaccinology

**DOI:** 10.1017/S003118202400043X

**Published:** 2024-08

**Authors:** José de la Fuente, Srikant Ghosh

**Affiliations:** 1 SaBio. Instituto de Investigación en Recursos Cinegéticos IREC-CSIC-UCLM-JCCM, Ronda de Toledo 12, 13005 Ciudad Real, Spain; 2 Department of Veterinary Pathobiology, Center for Veterinary Health Sciences, Oklahoma State University, Stillwater, OK 74078, USA; 3 Entomology Laboratory, Parasitology Division, ICAR-Indian Veterinary Research Institute, Izatnagar 243122, Bareilly, UP, India; 4 Eastern Regional Station- Indian Veterinary Research Institute, 37 Belgachia Road, Kolkata-700037, West Bengal, India

**Keywords:** adjuvant, subolesin, tick, tick-borne disease, vaccine, vaccinomics

## Abstract

Ticks represent a major concern for society worldwide. Ticks are also difficult to control, and vaccines represent the most efficacious, safe, economically feasible and environmentally sustainable intervention. The evolution of tick vaccinology has been driven by multiple challenges such as (1) Ticks are difficult to control, (2) Vaccines control tick infestations by reducing ectoparasite fitness and reproduction, (3) Vaccine efficacy against multiple tick species, (4) Impact of tick strain genetic diversity on vaccine efficacy, (5) Antigen combination to improve vaccine efficacy, (6) Vaccine formulations and delivery platforms and (7) Combination of vaccines with transgenesis and paratransgenesis. Tick vaccine antigens evolved from organ protein extracts to recombinant proteins to chimera designed by vaccinomics and quantum vaccinomics. Future directions will advance in these areas together with other novel technologies such as multiomics, AI and Big Data, mRNA vaccines, microbiota-driven probiotics and vaccines, and combination of vaccines with other interventions in collaboration with regions with high incidence of tick infestations and tick-borne diseases for a personalized medicine approach.

## Graphical abstract

**Figure:**
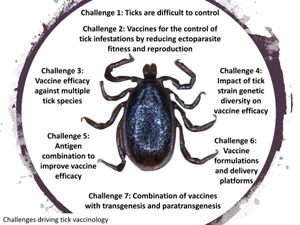

## Challenge 1: ticks are difficult to control

Ticks and tick-borne pathogens constitute a growing problem with increasing social and economic concern worldwide (e.g. de la Fuente *et al*., 2023*a*). Ticks are difficult to control, and traditional control methods are mainly based on the use of chemical acaricides with partial success and drawbacks such as selection of resistant ticks and negative impact on animal health and production and environmental contamination (Agwunobi *et al*., 2021; Githaka *et al*., 2022; Gonzaga *et al*., 2023). A number of reports of establishment of multiacaricides resistant ticks in different parts of the world (Bishop *et al*., 2023) and growing global public concern of environment pollution due to high use of chemical acaricides has posed serious challenges on continuation the use of conventional methods for tick management.

Under the One Health and sustainability perspective, vaccines are the most effective and safe intervention to reduce tick populations and risks associated with transmitted pathogens (de la Fuente, 2018; reviewed by Estrada-Peña *et al*., 2022). However, although a number of reports of significant efficacy of other vaccine formulations have been reported (de la Fuente and Kocan, 2003; de la Fuente and Contreras, 2015; Bishop *et al*., 2023; Parizi *et al*., 2023), only Bm86/Bm95-based vaccines TickGARD in Australia and Gavac in Cuba were registered and commercialized for the control of *Rhipicephalus microplus* tick infestations (de la Fuente *et al*., 2007; Rodríguez-Mallon, 2023). Currently, only Gavac (CIGB, Havana, Cuba; https://www.cigb.edu.cu/en/product/gavac-2/) and Bovimune Ixovac (Lapisa, La Piedad, Michoacán, Mexico; https://lapisa.com/productos/bovimune-ixovac) with Bm86 antigen are still commercially available in some Latin American countries.

**Figure 1.**
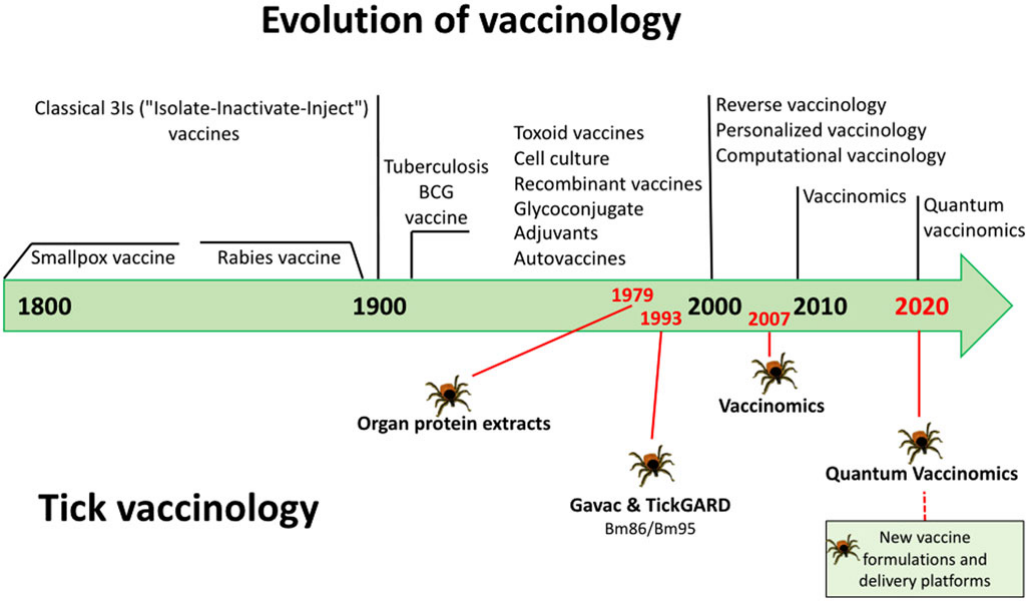
Tick vaccine research in the context of the evolution of vaccinology. Key advances in tick vaccinology are highlighted in red with tick stickers.

Based on the evolution of vaccinology (Andreano *et al*., 2019), this review approached the evolution of tick vaccinology to face challenges and advance in the development of new effective anti-tick vaccines and other control interventions (Fig. 1).

## Challenge 2: vaccines control tick infestations by reducing ectoparasite fitness and reproduction

The proof-of-concept of anti-tick vaccine was proposed by Allen and Humphreys (1979) using organ specific protein extracts. The first challenge was then approached with the discovery of *R. microplus* Bm86/Bm95 antigen and the development, registration and commercialization of TickGARD and Gavac vaccines for the control of cattle tick infestations (Willadsen *et al*., 1988, 1995; Rodríguez *et al*., 1994; reviewed by de la Fuente and Kocan, 2003; de la Fuente *et al*., 2007; Rodríguez-Mallon, 2023). The protective mechanism was associated with antibody production in response to vaccine and antibody-antigen interactions in the midgut lumen of ticks feeding on immunized host (Willadsen and Kemp, 1988). This interaction affected tick protein function, which translated into reduction in the number of ticks completing life cycle, weight, oviposition and fertility (de la Fuente and Kocan, 2014). Considering the role of cattle hosts in tick-borne diseases (TBD), these vaccines may not only reduce tick infestations and incidence of TBD in cattle but also in humans and other animal species (Chakraborty *et al*., 2023). However, due to significant variation in vaccine efficacy reported of 0–100% (de la Fuente and Kocan, 2014; Parizi *et al*., 2023) against different strains of *R. microplus*, these vaccines have not been approved in most countries.

## Challenge 3: vaccine efficacy against multiple tick species

Despite the advances on anti-tick *R. microplus* vaccines with Bm86/Bm95 antigens, conserved protective antigens across different tick genera needed to be identified. To address this challenge, Subolesin (SUB; originally named 4D8 and ortholog of Akirin) was discovered by expression library immunization in *Ixodes scapularis* mouse model (Almazán *et al*., 2003). The SUB-vaccine protective responses were not only mediated by anti-SUB antibodies entering tick cells by unknown mechanisms and blocking protein translocation to the nucleus to exert its regulatory function, but also through activation of other immune protective mechanisms (de la Fuente *et al*., 2011, 2021; Merino *et al*., 2011; Artigas-Jerónimo *et al*., 2020). The immune response to SUB affects multiple biological processes, which translates in various hosts (e.g. cattle, deer, sheep, dog, rabbit, mouse, chicken) into reduction of fitness and reproduction of different tick species (e.g. *Ornithodoros*, *Ixodes*, *Haemaphysalis*, *Amblyomma*, *Dermacentor*, *Hyalomma*, *Rhipicephalus*) and other arthropod vectors (e.g. mosquito, sand fly, poultry red mite) and vector-borne pathogens (e.g. *Anaplasma*, *Babesia*, *Borrelia*, *Plasmodium*) (Artigas-Jerónimo *et al*., 2018; Parizi *et al*., 2023) (Table 1). The efficacy and effectiveness of vaccines with SUB antigens have been evaluated not only under pen-controlled conditions (Shakya *et al*., 2014; Artigas-Jerónimo *et al*., 2018), but also in field trials (Torina *et al*., 2014; Mendoza-Martínez *et al*., 2021). Under field conditions in vaccinated cattle and sheep, the results showed 63% of sheep tick infestations, 8-fold reduction in the per cent of infested cattle, 32–55% reduction in tick weight, reduction in acaricide treatments and in the prevalence of *Anaplasma marginale* tick-transmitted genotypes (Torina *et al*., 2014). Recently, SUB vaccine provided a 67% efficacy in cattle infested with *R. microplus* (Mendoza-Martínez *et al*., 2021) and 83–90% efficacy in cattle vaccinated with *Rhipicephalus appendiculatus* SUB and infested with *R. appendiculatus*, *Rhipicephalus decoloratus* and *Amblyomma variegatum* (Kasaija *et al*., 2020).

**Table 1.** Examples of the efficacy of animal immunization with SUB tick protective antigen

Tick species	Expression system	Host	Dose and delivery	Adjuvant	Efficacy (E) against infestation (%)	Reference
*I. scapularis*	*E. coli*	Rabbit	500 *μ*g 2 doses; S/C	Freund's incomplete adjuvant	46%, *I. scapularis*	Almazán *et al*. (2005)
*R. microplus*	*P. pastoris*	Chicken	50 *μ*g 3 doses; S/C	Montanide ISA 50 V	35.1%, *Dermanyssus gallinae gallinae*	Harrington *et al*., 2009
*R. microplus*	*E. coli*	Cattle	100 *μ*g 3 doses; I/M	Montanide ISA 50 V	*R. annulatus* larvae, adults, 37–48%	Almazán *et al*. (2010)
*A. americanum*	*E .coli*	Cattle	100 *μ*m 3 doses; S/C	Montanide ISA 50 V	55%	de la Fuente *et al*., (2010)
*I. scapularis*	Vaccinia virus	Mice	10^8^ pfu given orally	No adjuvant used	52%	Bensaci *et al*., 2012
*R. microplus*	*E. coli*	White-tailed deer	100 *μ*g 3 doses; I/M	Montanide ISA 50 V	*83%*	Carreón, *et al*., 2012
R. microplus	*P. pastoris*	Mice	25 *μ*g 3 doses; I/M	Montanide ISA 50V2	*I. ricinus* larvae, 54%	Moreno-Cid *et al*., 2013
*R. microplus*	*E. coli*	Cattle	100 *μ*g 3 doses; I/M	Montanide ISA 50 V	60%	Merino *et al*., 2013
*R. microplus*	*E. coli*	Cattle	100 *μ*g 2 doses; I/M	Montanide 888	44%.	Shakya *et al*., 2014
*R. appendiculatus*	*E. coli*	Cattle	100 *μ*g 3 doses; I/M	Montanide ISA 50V2	*R. appendiculatus*, 47–90%; *A. variegatum,* 50–89%; *R. decoloratus,* 51%	Kasaija *et al*., 2020
*A. variegatum*	*E. coli*	Cattle	100 *μ*g 3 doses; I/M	Montanide ISA 50V2	*R. appendiculatus,* 83–86%; *A. variegatum,* 47–76%; *R. decoloratus,* 72%	Kasaija *et al*., 2020
*R. decoloratus*	*E. coli*	Cattle	100 *μ*g 3 doses; I/M	Montanide ISA 50V2	*R. appendiculatus* 66–89*%;* *A. variegatum* 50–89%; *R. decoloratus,* 51%	Kasaija *et al*., 2020
*H. longicornis*	*E.coli*	Rabbit	500 *μ*g 2 doses; I/M	TiterMax Gold	37.4%	Lee *et al*., 2020
*R. microplus* SUB peptide	*E. coli*	Cattle	100 *μ*g 3 doses; S/C	Montanide ISA 50	67%	Mendoza-Martínez *et al*., 2021
*R. microplus*	*E. coli*	Cattle	oral	Montanide ISA 50V2	*R. decoloratus,* 7196%; *R. appendiculatus,* 87–99%	Kasaija *et al*., 2022

Abbreviations: S/C, subcutaneous; I/M, intramuscular; pfu, plaque forming units.

Taken together, these results support the efficacy of SUB vaccines against different tick genera and other arthropod vector species. Additionally, other antigens such as p29, Aquaporin, Metalloprotease, Potassium ion channels, Protease inhibitors, Calreticulin, P0, Ferritin 2 and Tropomyosin have shown protection against different tick species (de la Fuente and Kocan, 2003; de la Fuente and Contreras, 2015; Manjunathachar *et al*., 2019; Abbas *et al*., 2023; Parizi *et al*., 2023; de la Fuente *et al*., 2023*b*; Nepveu-Traversy *et al*., 2024).

## Challenge 4: impact of tick strain genetic diversity on vaccine efficacy

Even if tick vaccine antigens such as SUB have shown efficacy against multiple tick species, the challenge related to strain genetic diversity and other factors needs to be considered. To face this challenge, a ‘personalized medicine’ approach was proposed considering regional, tick species/strains and host factors.

An example of this approach is the SUB antigen from *R. appendiculatus*, *R decoloratus* and *A. variegatum*, main tick species infesting *Bos indicus* and crossbred cattle in Uganda (Kasaija *et al*., 2020). Vaccine formulations with antigens from these tick species were evaluated under controlled pen conditions in both cattle breeds to select *R. appendiculatus*-derived SUB as the antigen with higher cross-species protection (Kasaija *et al*., 2020). This vaccine is now under field trial in Uganda (Kabi *et al*., 2022). Other personalized SUB vaccines have been evaluated against different Indian tick species (Parthasarathi *et al*., 2023).

These results highlight the importance of personalizing vaccines considering tick, host and livestock farm management factors to improve effectiveness under field conditions.

## Challenge 5: antigen combination to improve vaccine efficacy

Antigen combinations have been considered to improve vaccine efficacy and results of experimental trials provided support for this approach (e.g. Vitellin-degrading cysteine endopeptidase (VTDCE), *Boophilus* yolk pro-cathepsin (BYC) and Glutathione S-transferase (GST-Hl), Parizi *et al*., 2012; Bm86, SUB and Tropomyosin (TPM), Parthasarathi *et al*., 2023; Bm86 and P0 peptide, Rodríguez-Mallon *et al*., 2023) (Table 2). A comparatively higher efficacy was noted when compared with single antigen immunization. However, the main limitation of this approach is that protein-protein physical and immunological interactions may affect protective immune response in vaccinated hosts and thus additional experiments are required to eliminate the possible constraints in developing vaccine formulation using multiple antigens.

**Table 2.** Examples of the efficacy of vaccination of animals with SUB combined with other tick/parasite antigens

Antigen I	Antigen II	Antigen III	Host	Dose and delivery	Adjuvant	Efficacy	Reference
*R. microplus* BM86	*H. anatolicum* SUB	*H.anatolicum* tropomyosin (TPM)	Cattle	Each antigen 100 *μ*g 3 doses; I/M; Co-vaccination	Montanide ISA 50V2	87.2% and 86.2% against *H. anatolicum* larvae and adults; 86.7% against *R. microplus*	Parthasarathi *et al*., 2023
*R. microplus* BM86	SUB peptide of *R. microplus*	*----*	Cattle	100 *μ*g 3 doses; S/C; dual vaccine	Montanide ISA 50V2	49%	Mendoza-Martínez *et al*., 2021
*R. microplus* SUB	*Anaplasma marginale* Major surface protein-1	*----*	Cattle	120 *μ*g 3 doses; S/C; chimeric vaccine	Montanide ISA 50V2	81%	Almazán et al. (2012)
*R. appendiculatus* SUB	*A. variegatum* SUB	*R. decoloratus* SUB	Cattle	Cocktail of 100*μ*g each protein prepared 3 doses; S/C; Cocktail vaccine	Montanide ISA 50V2	*R. appendiculatus* 74–92; *A. variegatum* 51–69%; *R. decoloratus* 71%	Kasaija *et al*., 2020
*R. microplus SUB*	heat inactivated *Mycobacterium bovis* (IV)	*----*	Cattle	200 *μ*g of SUB mixed with 6 × 10^6^cfu IV in 18 ml PBS for SUB + IV 2 doses; Oral	IV acts as adjuvant	65%	Contreras *et al*. (2019*a*, 2019*b*)
Subolesin-Akirin chimera (Q38)		*-----*	Roe deer	100 *μ*g 3 dose I/M; chimeric vaccine	Montanide ISA 50V2	More than 95% against *I. ricinus* and 46.4% *Dermacentor reticulatus* larvae	Contreras *et al*., 2020
Protective epitopes of *I. scapularis* SUB	Protective epitopes of *A. albopictus* Akirin						
SUB-MSP1a construct		*----*	Cattle	100 *μ*g 2 doses; I/M; chimeric vaccine	Montanide ISA 50V2	60%	Almazán et al. (2012)
*R. microplus SUB*	*Anaplasma* *marginale* Major Surface Protein 1a (MSP1a)						

To approach this limitation, the possibility of combining SUB DNA and protein in a vaccine formulation was considered (Hassan *et al*., 2020). However, recent research has focused on quantum vaccinomics algorithms for the combination of antigen protective epitopes or immunological quantum (Artigas-Jerónimo *et al*., 2020; Contreras *et al*., 2022*a*, 2022*b*). As recently proposed (de la Fuente *et al*., 2023*b*), in this approach, the prediction, identification and validation of protective epitopes is based on the combination of *in vitro*, *in silico*, *in music* and epitope mapping approaches with systems biology integration of omics datasets, artificial intelligence (AI) and Big Data (Villar *et al*., 2017; de la Fuente *et al*., 2018; de la Fuente and Contreras, 2023).

Vaccinomics is based on the integrations of omics dataset for the identification of candidate vaccine protective antigens (Poland *et al*., 2013; de la Fuente and Merino, 2013; Contreras *et al*., 2016, 2017, 2019*a*). The proposal of quantum vaccinomics originated from vaccinomics and the random processes such as immunoglobulin recombination events, direct correlation between atomic coordination and peptide immunogenicity and quantum dynamics of the immune response that has been subjected to optimizing evolution within living organisms supporting quantum immunology (reviewed by de la Fuente and Contreras, 2021). Then, in reference to Albert Einstein quantum of light, immune protective epitopes were proposed as immunological quantum and quantum vaccinomics as the identification and combination of antigen immunological quantum for vaccine development (Artigas-Jerónimo *et al*., 2020).

Antigens such as Q38 with SUB protective epitopes (Artigas-Jerónimo *et al*., 2020; de la Fuente *et al*., 2023*b*) have shown protection against tick infestations and other arthropod vectors (Merino *et al*., 2013; Moreno-Cid *et al*., 2013; Contreras *et al*., 2020; Letinić *et al*., 2021) with correlation between SUB-reactive epitopes and vaccine efficacy (Contreras *et al*., 2022*a*). The chimeric antigen RmSEI composed of *R. microplus* Subtilisin inhibitor 7 (RmSI-7), a Trypsin inhibitory like serine protease inhibitor, an interdomain region from the Kunitz inhibitor BmTI-A, and a cysteine-rich AMP-like Microplusin (RmSEI) was designed and showed anti-tick and antimicrobial activities (Costa et al., 2023). This approach can also be used to combine tick with pathogen derived antigens (Shrivastava *et al*., 2020). Two multiepitopic peptides using amino acid sequences of ferritin-2 (FER2) and tropomyosin (TPM) vitellogenin receptor (VgR) were synthesized and tested against *H. anatolicum* infestations with more than 80% efficacy (Nandi *et al*., 2023) (Table 2).

Quantum vaccinomics also considers immune mechanisms mediated by protein post-translational modifications such as carbohydrate alpha-gal (Gal*α*1-3Gal*β*1-4GlcNAc) present in glycoproteins (Galili, 2021) to address limitations of reductionists methods such as reverse vaccinology (Van Regenmortel, 2018; de la Fuente *et al*., 2023*b*). Accordingly, quantum vaccinomics covers some of the proposed top biotechnology trends in 2024 (https://www.startus-insights.com/innovators-guide/top-10-biotech-industry-trends-innovations-in-2021/) including AI, Big Data, gene editing, precision medicine, gene sequencing, biomanufacturing and synthetic biology.

In this way, quantum vaccinomics for protective antigen design considers vaccine efficacy and safety, geographic, environmental and population factors, host-tick-pathogen interactions and derived factors and host immunity for vaccinomics and adversomics.

## Challenge 6: vaccine formulations and delivery platforms

Even when protective antigens are identified or designed, formulations and delivery are the key components of vaccine efficacy. Regarding tick control, recent advances in vaccine formulations targeting vector gut microbiota commensal bacteria was found effective (Mateos-Hernández *et al*., 2020, 2021). Experimental manipulation of the microbiota has been achieved by antibiotic exposure or sterile-rearing conditions of the vector. Anti-microbiota vaccine impacted tick physiology by increasing tick weight during feeding and modulated tick microbiota composition and diversity in a taxon-specific manner. The impact of anti-microbiota vaccines on pathogen development was shown in *Plasmodium relictum* and the mosquito vector *Culex quinquefasciatus* (Aželytė *et al*., 2022), and recently it was reported that perturbations of tick microbiota can impact highly sensitive *Borrelia* spp. with departure from the modulation induced by the pathogen in the vector microbiota posing a high cost to the spirochete (Wu-Chuang *et al*., 2021). However, these methods induce global changes in the microbiota and make the depletion of specific bacteria difficult. Recently, anti-microbiota vaccines were proposed as a precise tool for microbiota manipulation (Wu-Chuang *et al*., 2021; Maitre *et al*., 2022). Other advances including probiotics and formulations with high alpha-gal content (Cabezas-Cruz and de la Fuente, 2017; Hodžić *et al*., 2020; Bamgbose *et al*., 2021) and adjuvants with heat-inactivated alpha-gal-containing bacteria for oral vaccine administration (Contreras *et al*., 2019*b*; Kasaija *et al*., 2022). Oral vaccine formulations combining *R. appendiculatus*-derived SUB with heat-inactivated mycobacteria resulted in 96% and 99% efficacy against *R. decoloratus* and *R. appendiculatus*, respectively (Kasaija *et al*., 2022).

Tick vaccines have mainly been designed with recombinant antigens, but recent research includes advances in mRNA vaccines (Sajid *et al*., 2021; Boulanger and Wikel, 2023; Matias *et al*., 2023). For antigen combination, chimeric antigens on microparticles and mRNA-lipid nanoparticles may be considered for vaccine delivery (Sajid *et al*., 2021; Matias *et al*., 2023).

## Challenge 7: combination of vaccines with transgenesis and paratransgenesis

Recently, Cas9-mediated gene editing was implemented in ticks by embryo injection and ReMOT Control (Sharma *et al*., 2022). The CRISPR-Cas molecular machines also provide interventions for paratransgenesis to manipulate tick microbiome and virome composition and function (Ramachandran and Bikard, 2019).

More recently, Frankenbacteriosis was developed for paratransgenic manipulation of tick commensal *Sphingomonas* bacterium to reduce tick fitness and *Anaplasma phagocytophilum* pathogen infection (Mazuecos *et al*., 2023*a*, 2023*b*; de la Fuente *et al*., 2023*b*).

Transgenesis and paratransgenesis may be combined with anti-tick vaccines and other control interventions including the proposed Suicidalbacteriosis in which tick commensal bacteria are manipulated to produce and secrete antigens protective against ticks and tick-borne pathogens to immunize hosts during blood feeding (de la Fuente *et al*., 2023*b*). For example, genetic manipulation of tick microbiome and virome composition and function may produce ticks more susceptible to tick vaccine induced host immune response thus improving vaccine efficacy for the control of tick infestations and vector capacity.

However, application of gene editing technology involves risks since it may produce off target deleterious mutations. A high frequency of off-target effects has been reported in human cells but low in mice and zebrafish (Hwang *et al*., 2013; Yang *et al*., 2013). Large genomes may contain identical or homologous DNA sequences to intended target DNA sequence. Gene editing technology may delete these unintended sequences causing mutations which may cause cell death or transformation. Efforts have been made to reduce off-target mutations, but further improvement is required. Another problem is efficient safe delivery of CRISPR-Cas9 into cell types that are hard to transfect. If there is a risk of transferring genes to other species, there is risk of transferring modified sequences. It is difficult to control dispersion of gene driven trait. Moreover, disappearance of whole populations targeted by gene drive may have serious consequences in the ecosystem equilibrium. All these risk factors demand careful assessment of each potential application and need for critical regulatory norms.

## Conclusions and future directions

Tick vaccine antigens evolved from organ specific protein extracts to recombinant proteins to vaccinomics algorithms for designing chimeric antigens. Recent advances in tick vaccinology and future directions include discovery of novel protective antigens (de la Fuente and Contreras, 2015; Abbas *et al*., 2023) including the application of AI and Big Data analytic techniques (de la Fuente *et al*., 2018), novel vaccine formulations and delivery platforms (Ndawula, 2021; Tabor, 2021; Pereira *et al*., 2022), mRNA vaccines (Sajid *et al*., 2021; Matias *et al*., 2023; Boulanger and Wikel, 2023), vaccinomics and quantum vaccinomics (Poland *et al*., 2013; de la Fuente and Contreras, 2021, 2023; Contreras *et al*., 2022*b*). Other methods include use of formulations with combined protective antigens (Ndawula and Tabor, 2020; Parthasarathi *et al*., 2021), probiotics and other formulations targeting tick microbiota (Cabezas-Cruz and de la Fuente, 2017; Hodžić *et al*., 2020; Mateos-Hernández *et al*., 2020, 2021; Wu-Chuang *et al*., 2023). To improve vaccine efficacy, post-translational modifications such as alpha-gal have also been considered to improve vaccine efficacy (Hodžić *et al*., 2020). Moreover, characterization of tick-host-pathogen interactions, immune protective and acaricide-resistance mechanisms (Bhowmick and Han, 2020; Bishop *et al*., 2023; Waldman *et al*., 2023), transgenesis and paratransgenesis for the genetic manipulation of commensal bacteria and ticks (Sharma *et al*., 2022; Mazuecos *et al.* 2023*a*; de la Fuente *et al*., 2023*b*) and combination of vaccines with other interventions such as natural plant and animal-derived compounds and cultural practices among other interventions (Showler and Saelao, 2022) were considered as possible alternatives. International collaborations with regions with high incidence of tick infestations and TBD (Estrada-Peña and de la Fuente, 2023), personalized medicine approach based on regional, tick species/strains and host-driven variables (Kasaija *et al*., 2020) are also proposed for sustainable management of the relevant vector.

## Data Availability

All data used in the study is disclosed in the paper and corresponding references.
